# Cardiac power output is associated with adverse outcomes in patients with preserved ejection fraction after transcatheter aortic valve implantation

**DOI:** 10.1093/ehjimp/qyae048

**Published:** 2024-05-21

**Authors:** Daisuke Miyahara, Masaki Izumo, Yukio Sato, Tatsuro Shoji, Mitsuki Yamaga, Yoshikuni Kobayashi, Takahiko Kai, Taishi Okuno, Shingo Kuwata, Masashi Koga, Yasuhiro Tanabe, Yoshihiro J Akashi

**Affiliations:** Department of Cardiology, St. Marianna University School of Medicine, 2-16-1, Sugao, Miyamae-ku, Kawasaki 216-8511, Japan; Department of Cardiology, St. Marianna University School of Medicine, 2-16-1, Sugao, Miyamae-ku, Kawasaki 216-8511, Japan; Department of Cardiology, St. Marianna University School of Medicine, 2-16-1, Sugao, Miyamae-ku, Kawasaki 216-8511, Japan; Department of Cardiology, St. Marianna University School of Medicine, 2-16-1, Sugao, Miyamae-ku, Kawasaki 216-8511, Japan; Department of Cardiology, St. Marianna University School of Medicine, 2-16-1, Sugao, Miyamae-ku, Kawasaki 216-8511, Japan; Department of Cardiology, St. Marianna University School of Medicine, 2-16-1, Sugao, Miyamae-ku, Kawasaki 216-8511, Japan; Department of Cardiology, St. Marianna University School of Medicine, 2-16-1, Sugao, Miyamae-ku, Kawasaki 216-8511, Japan; Department of Cardiology, St. Marianna University School of Medicine, 2-16-1, Sugao, Miyamae-ku, Kawasaki 216-8511, Japan; Department of Cardiology, St. Marianna University School of Medicine, 2-16-1, Sugao, Miyamae-ku, Kawasaki 216-8511, Japan; Department of Cardiology, St. Marianna University School of Medicine, 2-16-1, Sugao, Miyamae-ku, Kawasaki 216-8511, Japan; Department of Cardiology, St. Marianna University School of Medicine, 2-16-1, Sugao, Miyamae-ku, Kawasaki 216-8511, Japan; Department of Cardiology, St. Marianna University School of Medicine, 2-16-1, Sugao, Miyamae-ku, Kawasaki 216-8511, Japan

**Keywords:** cardiac power output, transcatheter aortic valve implantation, aortic stenosis, preserved left ventricular ejection fraction

## Abstract

**Aims:**

Cardiac power output (CPO) measures cardiac performance, and its prognostic significance in heart failure with preserved ejection fraction (EF) has been previously reported. However, the effectiveness of CPO in risk stratification of patients with valvular heart disease and post-operative valvular disease has not been reported. We aimed to determine the association between CPO and clinical outcomes in patients with preserved left ventricular (LV) EF after transcatheter aortic valve implantation (TAVI).

**Methods and results:**

This retrospective observational study included 1047 consecutive patients with severe aortic stenosis after TAVI. All patients were followed up for all-cause mortality and hospitalization for HF. CPO was calculated as 0.222 × cardiac output × mean blood pressure (BP)/LV mass, where 0.222 was the conversion constant to W/100 g of the LV myocardium. CPO was assessed using transthoracic echocardiography at discharge after TAVI. Of the 1047 patients, 253 were excluded following the exclusion criteria, including those with low LVEF, and 794 patients (84.0 [80.0–88.0] years; 35.8% male) were included in this study. During a median follow-up period of 684 (237–1114) days, the composite endpoint occurred in 196 patients. A dose-dependent association was observed between the CPO levels and all-cause mortality. Patients in the lowest CPO tertile had significantly lower event-free survival rates (log-rank test, *P* = 0.043). Multivariate Cox regression analysis showed that CPO was independently associated with adverse outcomes (hazard ratio = 0.561, *P* = 0.020). CPO provided an incremental prognostic effect in the model based on clinical and echocardiographic markers (*P* = 0.034).

**Conclusion:**

CPO is independently and incrementally associated with adverse outcomes in patients with preserved LVEF following TAVI.

## Graphical Abstract

**Graphical Abstract:**
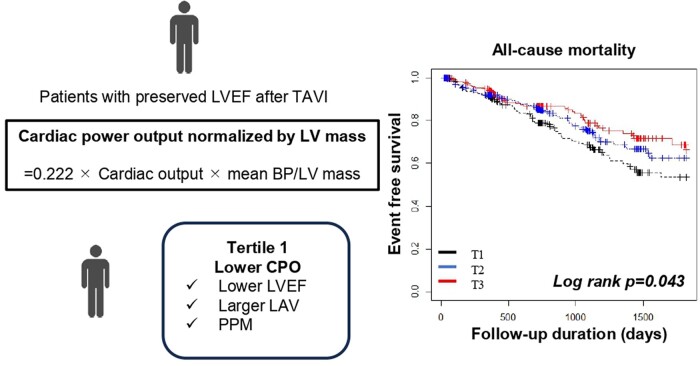

## Introduction

Transcatheter aortic valve implantation (TAVI) is an established therapy for symptomatic severe aortic stenosis (AS).^1–3^ However, mortality and heart failure readmission after TAVI are common.^4–6^ A previous study showed that reduced left ventricular ejection fraction (LVEF) is associated with an increased risk of poor outcomes in patients with AS after TAVI.^7^ However, some patients with preserved LVEF after TAVI exhibit poor prognosis, a phenomenon that has been associated with heart failure with preserved EF (HFpEF).^8^

Cardiac power output (CPO) measures cardiac performance^9^ and is calculated as the product of the energy work generated by the LV multiplied by the heart rate (HR) based on the pressure–volume loop. A lower CPO, which is influenced by various factors such as LV contractility, preload, afterload, and right ventricular function, is associated with adverse clinical outcomes in patients with HFpEF.^10,11^ In addition, a previous study showed that CPO to LV mass was a better measure of myocardial performance, because it reflects the power-generating capacity per unit mass of LV myocardium.^12^ However, whether lower CPO drives poor outcomes or is a surrogate for disease severity remains to be established. Previous studies examining the relationship between CPO and prognosis did not include patients with valvular heart disease or post-operative valvular disease, and the effectiveness of CPO in risk stratification after TAVI has not been reported. Therefore, we performed a retrospective analysis to evaluate CPO in patients with preserved LVEF after TAVI. We hypothesize that a lower CPO would be associated with worse clinical outcomes in patients with preserved LVEF after TAVI.

## Methods

### Study design

This retrospective observational study included 1047 consecutive patients who underwent TAVI for severe AS between January 2017 and July 2023 at St. Marianna University Hospital, Kawasaki, Japan. The study protocol was approved by the Ethics Committee of St. Marianna University School of Medicine (No. 6336), and the need for informed consent was waived due to the retrospective nature of the study.

### Resting echocardiography

All patients underwent comprehensive two-dimensional and Doppler transthoracic echocardiography according to the guidelines of the American Society of Echocardiography^13^ before and within 1 week after TAVI. An experienced sonographer performed all echocardiographic procedures using a commercially available ultrasound system (Philips Healthcare, Andover, MA, USA, or General Electric Healthcare, Little Chalfont, UK). LV end-diastolic volume (EDV) and end-systolic volume (ESV) were measured using Simpson’s biplane method. LVEF was calculated as follows: [(EDV − ESV)/EDV] × 100. Continuous-wave Doppler was used to measure the maximal aortic valve velocities in apical three- or five-chamber views, and the mean gradients were estimated based on the Simplified Bernoulli equation. The LV outflow tract (LVOT) diameter was measured using zoomed parasternal long-axis views. The LVOT velocities acquired using pulsed-wave Doppler and velocity–time integrals (VTI LVOT) were measured. The stroke volume (SV) was calculated using the following formula: (LVOT diameter/2)^2^ × 3.14 × VTI LVOT. Subsequently, the SV index (SVi) and cardiac output (CO) were estimated using the following formulas: SVi = SV/body surface area (BSA) and CO = SV × HR. The CPO was then assessed non-invasively. Since power depends on the muscle volume-generating power, CPO was normalized using LV mass. CPO was calculated as 0.222 × CO × mean blood pressure (BP)/LV mass, where 0.222 was the conversion constant to W/100 g of the LV myocardium. The effective orifice area (EOA) was calculated using the continuity equation, and the EOA index was calculated by dividing by the BSA. Prosthesis-patient mismatch (PPM) was defined as an EOA index of ≤0.85 cm^2^/m^2^. Especially, severe PPM defined as an EOA index of ≤0.60 cm^2^/m^2^. Systolic pulmonary artery pressure (SPAP) was derived from the tricuspid regurgitation peak gradients by adding the estimated right atrial pressure from the inferior vena cava. The tricuspid annulus plane systolic excursion was assessed using M-mode on the tricuspid annulus and expressed as the longitudinal systolic function of shortening the right ventricle.

### Endpoint

Follow-up data were collected from the medical records. The primary endpoint of this study was defined as the time to the first occurrence of the composite endpoint comprising all-cause mortality and hospitalization for heart failure.

### Statistical analysis

Continuous variables were expressed as mean values with standard deviations or median values with interquartile ranges (IQR). Categorical variables were expressed as numbers and percentages. The Student’s *t*-test was used to analyse continuous variables with normal distributions, and the Mann–Whitney *U* test was used to analyse continuous variables with non-normal distributions. Categorical variables were compared using Fisher’s exact or χ^2^ tests, as appropriate.

Statistical significance was set at *P* < 0.05. The cumulative probability of event-free survival was estimated using the Kaplan–Meier method and compared between groups using a log-rank test. Univariable and multivariable Cox proportional hazard models were used to calculate hazard ratios (HRs) and 95% confidence intervals (CIs) for the clinical outcomes. The incremental prognostic effect of CPO was assessed using the likelihood ratio test by comparing models with and without the variables. Data analyses were performed using JMP 17 (SAS Institute Japan Inc., Tokyo, Japan) and R statistical software (version 4.2.2; R Foundation for Statistical Computing, Vienna, Austria).

## Results

**Figure 1:**
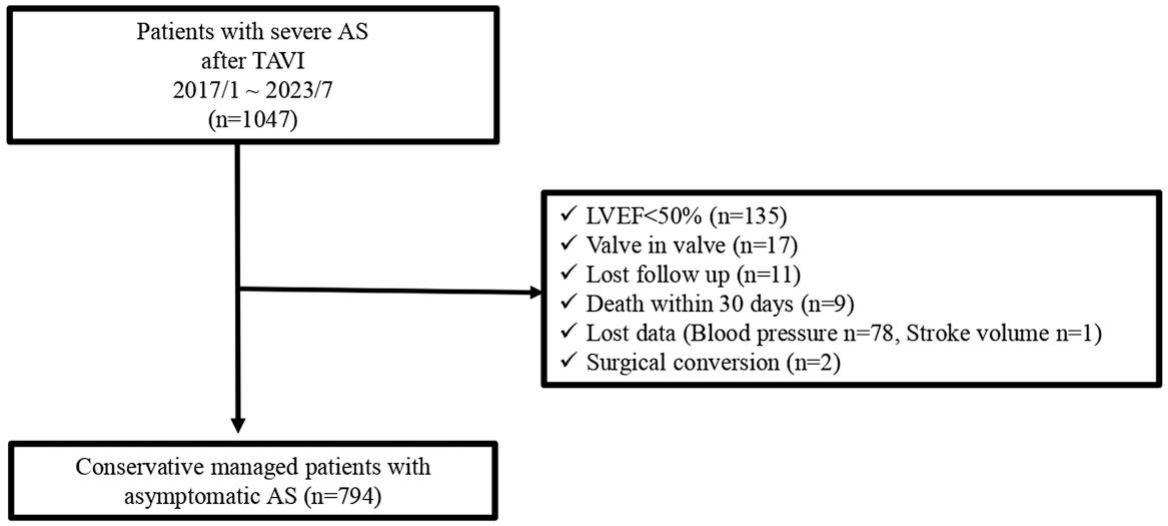
Flow chart showing study participant distribution.

A total of 253 patients were excluded for the following reasons: (i) low LVEF (<50%) (*n* = 135), (ii) valve in valve TAVI (*n* = 17), (iii) lost to follow-up (*n* = 11), (iv) death within 30 days (*n* = 9), (v) lost data [*n* = 79; blood pressure (*n* = 78) and stroke volume (*n* = 1)], and (vi) surgical conversion (*n* = 2). The remaining 794 patients were included in this study (*Figure 1*).

### Baseline characteristics

*Table 1* shows the baseline characteristics of the study cohort. Of the study cohort, 284 (35.8%) were male, with a median age of 84.0 (IQR, 80.0–88.0) years. The 794 patients were divided into three groups based on the tertile CPO value (tertile 1: <0.658 W/100 g; tertile 2: 0.658–0.929 W/100 g; and tertile 3: >0.929 W/100 g). The lower CPO group had significantly higher BSA, a higher percentage of males and smokers, higher cases of haemodialysis and atrial fibrillation (AF), higher prescription rate of mineralocorticoid receptor antagonist, lower estimated glomerular filtration rate (eGFR), and higher N-terminal pro-B-type natriuretic peptide (NT-pro BNP) levels. Age, other comorbidities, prescription rate of other medicines, and Hb levels did not differ among the three groups.

**Table 1:** Baseline clinical characteristics of the population

Variables	All (*n* = 794)	CPO tertile 1 (*n* = 264)	CPO tertile 2 (*n* = 265)	CPO tertile 3 (*n* = 265)	*P* value
Sex, male	284 (35.8)	118 (44.7)	92 (34.7)	74 (27.9)	<0.001
Age, years	84.0 (80.0–88.0)	84.0 (79.8–88.0)	84.0 (80.0–88.0)	84.0 (80.0–87.0)	0.295
BSA, m^2^	1.47 (1.35–1.60)	1.50 (1.40–1.65)	1.47 (1.35–1.59)	1.46 (1.34–1.58)	0.026
Hypertension	673 (84.8)	228 (86.4)	228 (86.0)	217 (81.9)	0.279
DM	226 (28.5)	78 (29.6)	70 (26.4)	78 (29.5)	0.664
CAD	163 (20.5)	51 (19.3)	60 (22.6)	52 (19.6)	0.578
Smoke	244 (30.7)	101 (38.3)	78 (29.4)	65 (24.5)	0.002
Haemodialysis	51 (6.4)	26 (9.8)	15 (5.7)	10 (3.8)	0.014
AF	168 (21.2)	74 (28.0)	58 (21.9)	36 (13.6)	<0.001
Medication					
β-Blocker	314 (39.6)	113 (42.8)	104 (39.2)	97 (36.6)	0.343
ACE inhibitor/ARB	428 (53.9)	146 (55.3)	151 (57.0)	131 (49.4)	0.187
ARNI	21 (2.6)	8 (3.0)	6 (2.3)	7 (2.6)	0.860
MRA	145 (18.3)	59 (22.3)	51 (19.2)	35 (13.2)	0.022
Ca channel blocker	512 (64.4)	172 (65.2)	172 (65.2)	168 (63.4)	0.915
PM	112 (14.1)	33 (12.5)	35 (13.2)	44 (16.6)	0.349
Hb, g/dL	10.8 (10.0–11.8)	11.0 (10.1–12.0)	10.8 (9.9–11.6)	10.7 (10.0–11.7)	0.409
eGFR, mL/min/1.73 m^2^	56.9 (41.9–71.0)	52.6 (38.2–67.7)	57.6 (42.2–68.8)	59.1 (45.3–75.8)	<0.001
NT-pro BNP, pg/mL (*n* = 760)	1307.5 (577.5–3071.8)	1758.5 (803.3–4569.3)	1340.0 (615.0–3352.0)	929.0 (365.5–2213.0)	<0.001
THV					0.075
BEV	650 (81.9)	206 (78.0)	217 (81.9)	227 (85.7)	
SEV	144 (18.1)	58 (22.0)	48 (18.1)	38 (14.3)	

Data are mean ± SD, median (IQR), or *n* (%).

BSA, body area; DM, diabetes mellitus; CAD, coronary artery disease; AF, atrial fibrillation; ACEi, angiotensin-converting enzyme inhibitor; ARB, angiotensin II receptor; ARNI, angiotensin receptor neprilysin inhibitor; MRA, mineralocorticoid receptor antagonist; PM, pacemaker; Hb, haemoglobin; eGFR, estimated glomerular filtration rate; THV, transcatheter heart valve; BEV, balloon-expandable valve; SEV, self-expandable valve.

### Echocardiography findings

Echocardiographic data after TAVI are summarized in *Table 2*. The lower CPO group had significantly lower systolic BP, HR, EOA, SV, CO, and LVEF, larger LV size and left atrial volume (LAV), higher LV mass and SPAP, and a higher rates of PPM, MR (≥3°), and TR (≥3°).

**Table 2:** Echocardiographic data of the population

Variables	All (*n* = 794)	CPO tertile 1 (*n* = 264)	CPO tertile 2 (*n* = 265)	CPO tertile 3 (*n* = 265)	*P* value
SBP, mmHg	132.3 (±21.5)	122.7 (±19.6)	132.9 (±20.0)	141.1 (±20.9)	<0.001
HR, beats/min	70.0 (62.8–79.0)	65.0 (58.8–72.0)	70.0 (63.0–77.0)	76.0 (69.0–85.0)	<0.001
EOA, cm^2^	1.54 (1.32–1.89)	1.48 (1.24–1.75)	1.53 (1.32–1.88)	1.62 (1.37–2.01)	<0.001
EOA index, cm^2^/m^2^	1.05 (0.89–1.27)	0.98 (0.86–1.12)	1.05 (0.89–1.28)	1.12 (0.95–1.36)	<0.001
PPM	132 (16.6)	58 (22.0)	44 (16.9)	28 (10.7)	0.002
Severe PPM	15 (1.9)	8 (3.0)	4 (1.5)	3 (1.1)	0.249
MPG, mmHg	10.0 (7.7–13.0)	9.7 (7.4–12.3)	10.2 (7.7–13.0)	10.1 (8.0–13.0)	0.229
LVDd, mm	42.0 (39.0–46.0)	45.0 (41.0–49.0)	43.0 (40.0–46.0)	40.0 (37.0–43.0)	<0.001
LVDs, mm	26.0 (24.0–30.0)	29.0 (26.0–32.0)	27.0 (24.0–30.0)	24.0 (22.0–27.0)	<0.001
LV mass index, g/m^2^	97.4 (82.2–116.1)	115.7 (101.2–135.2)	99.4 (87.1–111.2)	82.4 (71.3–92.6)	<0.001
SV, mL	66.0 (55.0–78.0)	61.0 (51.0–71.0)	66.0 (56.0–76.0)	73.0 (62.0–88.0)	<0.001
SV index, mL/m^2^	44.8 (38.3–51.9)	41.0 (34.2–46.7)	44.4 (38.7–51.0)	50.0 (43.5–58.6)	<0.001
CO, L/min	4.6 (3.9–5.5)	3.9 (3.3–4.6)	4.6 (3.9–5.3)	5.6 (4.8–6.5)	<0.001
CPO, W/100 g	0.771 (0.598–1.029)	0.520 (0.440–0.600)	0.770 (0.710–0.840)	1.190 (1.030–1.410)	<0.001
LVEDV, mL	80.0 (65.0–100.0)	90.0 (71.0–113.3)	82.0 (65.0–100.0)	74.0 (62.0–89.0)	<0.001
LVESV, mL	29.0 (22.0–39.0)	33.0 (25.0–44.0)	30.0 (22.0–38.0)	25.0 (20.0–32.0)	<0.001
LVEF, %	64.0 (59.8–68.0)	62.0 (58.0–66.0)	64.0 (60.0–68.0)	66.0 (62.0–69.0)	<0.001
LAD, mm	41.0 (36.0–46.0)	44.0 (40.0–50.0)	40.0 (36.0–45.5)	38.0 (34.0–43.0)	<0.001
LAV index, mL/m^2^	51.0 (39.4–65.4)	59.9 (47.2–74.1)	49.9 (40.1–64.2)	43.5 (34.3–56.0)	<0.001
E, cm/s	85.5 (66.0–108.0)	89.0 (71.0–114.0)	84.0 (67.0–108.0)	83.0 (64.0–106.0)	0.068
e′, cm/s	4.6 (3.8–5.6)	4.7 (3.7–5.8)	4.6 (3.6–5.4)	4.7 (3.8–5.6)	0.342
E/e′	18.0 (14.0–24.6)	18.5 (14.5–24.9)	17.8 (14.1–24.6)	17.6 (13.6–23.1)	0.092
E/A	0.67 (0.57–0.83)	0.74 (0.60–0.96)	0.68 (0.58–0.83)	0.64 (0.54–0.75)	<0.001
Abnormal	536 (85.2)	139 (76.0)	183 (85.1)	214 (93.0)	
Pseudonormal	93 (14.8)	44 (24.0)	33 (14.9)	16 (7.0)	
SPAP, mmHg	27.6 (22.9–32.4)	28.4 (23.6–35.4)	27.3 (22.9–32.2)	27.0 (22.5–31.7)	0.015
TAPSE, mm	18.9 (16.9–21.8)	18.5 (15.7–21.2)	19.3 (17.5–22.4)	19.3 (17.5–21.8)	0.085
PVL ≥ 2°	48 (6.1)	22 (8.3)	14 (5.3)	12 (4.5)	0.151
MR ≥ 3°	59 (7.4)	32 (12.1)	16 (6.0)	11 (4.2)	0.001
TR ≥ 3°	65 (8.2)	36 (13.6)	12 (4.5)	17 (6.4)	<0.001

Data are mean ± SD, median (IQR), or *n* (%).

SBP, systolic blood pressure; HR, heart rate; EOA, effective orifice area; PPM, prosthesis-patient mismatch; MPG, mean pressure gradient; LVDd, left ventricular diastolic dimension; LVDs, left ventricular systolic dimension; SV, stroke volume; CO, cardiac output; CPO, cardiac power output; LVEDV, left ventricular end-diastolic volume; LVESV, left ventricular end-systolic volume; LVEF, left ventricular ejection fraction; LAD, left atrial dimension; LAV, left atrial volume; SPAP, systolic pulmonary artery pressure; TAPSE, tricuspid annular plane systolic excursion; PVL, paravalvular leakage; MR, mitral regurgitation; TR, tricuspid regurgitation.

### Outcome analysis

**Figure 2:**
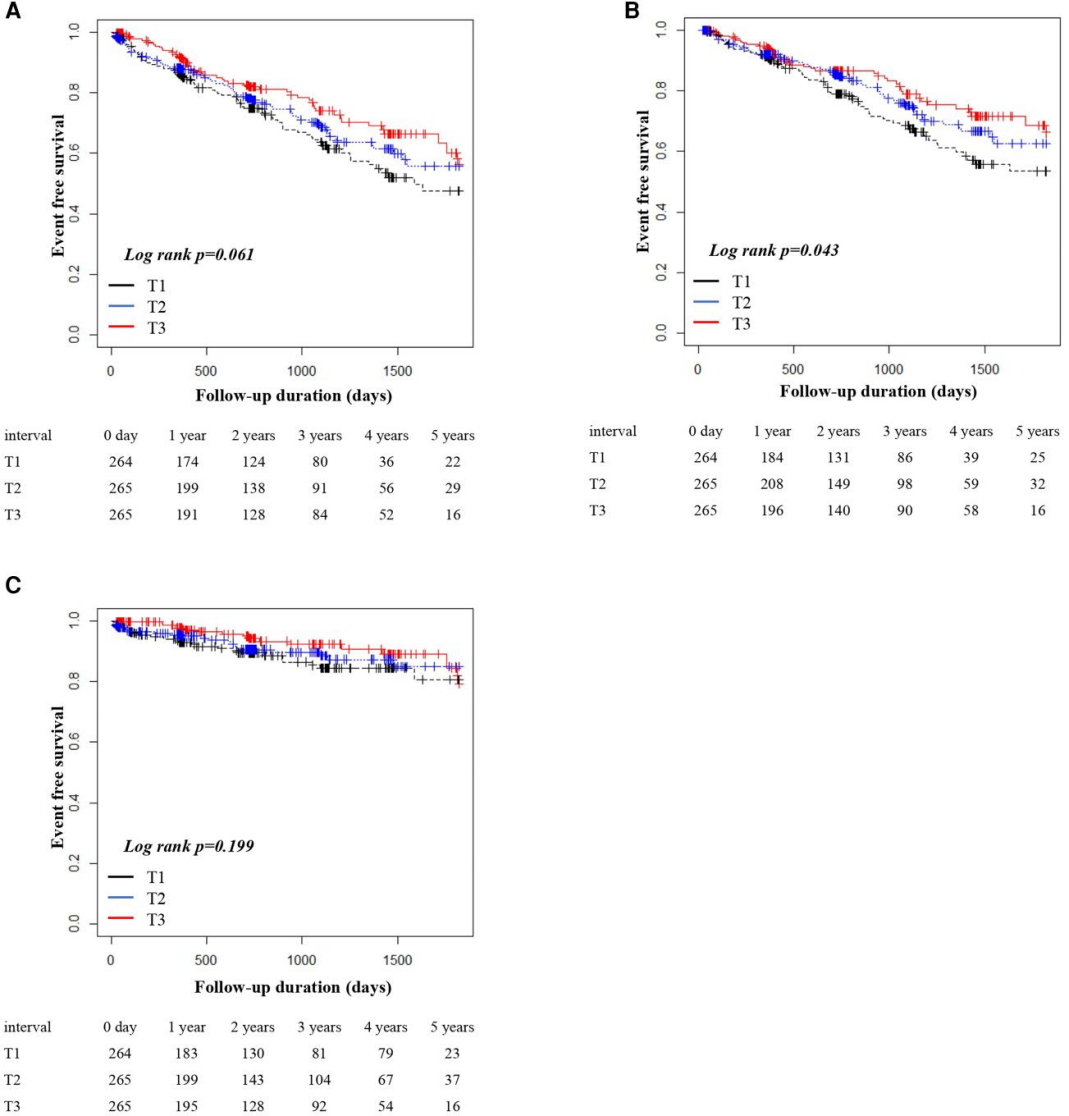
Kaplan–Meier survival curves for a composite of all-cause mortality or heart failure (HF) hospitalization. (*A*) Composite endpoints (*P* = 0.061). (*B*) All-cause mortality (*P* = 0.043). (*C*) Heart failure hospitalization (*P* = 0.199).

During a median follow-up of 684 days (IQR, 237–1114 days), the composite endpoint occurred in 196 patients (all-cause mortality, *n* = 160; hospitalization for heart failure, *n* = 66). Kaplan–Meier analysis revealed that for the composite endpoint, event-free survival tended to be lower in patients in the lowest tertile of CPO (log-rank test, *P* = 0.061; *Figure 2A*), and there was a dose-dependent association between CPO and all-cause mortality (log-rank test, *P* = 0.043; *Figure 2B*). However, there was no significant difference in hospitalization for heart failure among the groups (log-rank test, *P* = 0.199).

Univariable Cox regression analysis revealed that lower CPO and SV, higher SPAP, TR (≥3°), and AF were significantly associated with an increased risk of adverse events using the continuous variable format (*Table 3*). After adjusting for age, sex, and BSA, the same factors were found to be significantly associated with an increased risk of adverse events. Multivariable Cox regression analysis revealed that lower CPO, larger LAV, higher SPAP, and AF were independently associated with event risk (HR = 0.561, 0.992, 1.031, and 1.545, *P* = 0.020, 0.021, <0.001, and *P* = 0.031, respectively; *Table 3*).

**Table 3:** Uni- and multivariate Cox regression analyses for predicting composite endpoints in patients with preserved LVEF after TAVI

	Univariate model		Age-, BSA-, and gender-adjusted model		Multivariable model	
Variables	HR (95% CI)	*P* value	HR (95% CI)	*P* value	HR (95% CI)	*P* value
CPO, W/100 g	0.566 (0.374–0.855)	0.007	0.593 (0.392–0.896)	0.013	0.561 (0.345–0.913)	0.020
SV. mL	0.989 (0.981–0.997)	0.006	0.99 (0.983–0.999)	0.036	0.994 (0.985–1.003)^a^	0.198
CO, L/min	0.916 (0.824–1.017)	0.101	0.952 (0.860–1.054)	0.345	0.978 (0.876–1.093)^b^	0.695
LVM	1.002 (0.998–1.005)	0.313	1.003 (1.000–1.007)	0.058	1.005 (1.000–1.009)^c^	0.042
LVEF, %	0.982 (0.961–1.004)	0.111	0.979 (0.958–1.001)	0.062	0.983 (0.960–1.007)	0.158
E/e′	1.012 (0.996–1.028)	0.140	1.012 (0.995–1.029)	0.158	1.009 (0.989–1.030)	0.361
LAV, mL	1.002 (0.998–1.006)	0.338	1.003 (0.999–1.007)	0.157	0.992 (0.986–0.999)	0.021
SPAP, mmHg	1.033 (1.020–1.045)	<0.001	1.032 (1.019–1.045)	<0.001	1.031 (1.013–1.049)	<0.001
PM	0.740 (0.482–1.134)	0.167	0.784 (0.511–1.204)	0.267	0.760 (0.466–1.238)	0.270
PPM	0.879 (0.622–1.243)	0.467	0.985 (0.694–1.400)	0.934	0.823 (0.563–1.205)	0.317
PVL ≥ 2°	1.491 (0.918–2.422)	0.107	1.260 (0.772–2.056)	0.356	1.365 (0.818–2.278)	0.234
MR ≥ 3°	1.328 (0.817–2.157)	0.252	1.271 (0.782–2.066)	0.333	0.941 (0.542–1.632)	0.828
TR ≥ 3°	1.679 (1.095–2.575)	0.017	1.564 (1.018–2.402)	0.041	1.155 (0.673–1.984)	0.601
AF	1.483 (1.088–2.022)	0.013	1.587 (1.160–2.172)	0.004	1.545 (1.039–2.295)	0.031

Multivariable model was adjusted for age, sex, BSA, CPO, LVEF, E/e′, SPAP, PM, PPM, PVL, MR, TR, and AF.

BSA, body size; CPO, cardiac power output; SV, stroke volume; CO, cardiac output; LVM, left ventricular mass; LVEF, left ventricular ejection fraction; LAV, left atrial volume; SPAP, systolic pulmonary artery pressure; PM, pacemaker; PPM, prosthesis-patient mismatch; PVL, paravalvular leakage; MR, mitral regurgitation; TR, tricuspid regurgitation; AF, atrial fibrillation; TAVI, transcatheter aortic valve implantation.

^a^Model was adjusted for age, sex, BSA, SV, LVEF, E/e′, SPAP, PM, PPM, PVL, MR, TR, and AF.

^b^Model was adjusted for age, sex, BSA, CO, LVEF, E/e′, SPAP, PM, PPM, PVL, MR, TR, and AF.

^c^Model was adjusted for age, sex, BSA, LVM, LVEF, E/e′, SPAP, PM, PPM, PVL, MR, TR, and AF.

**Figure 3:**
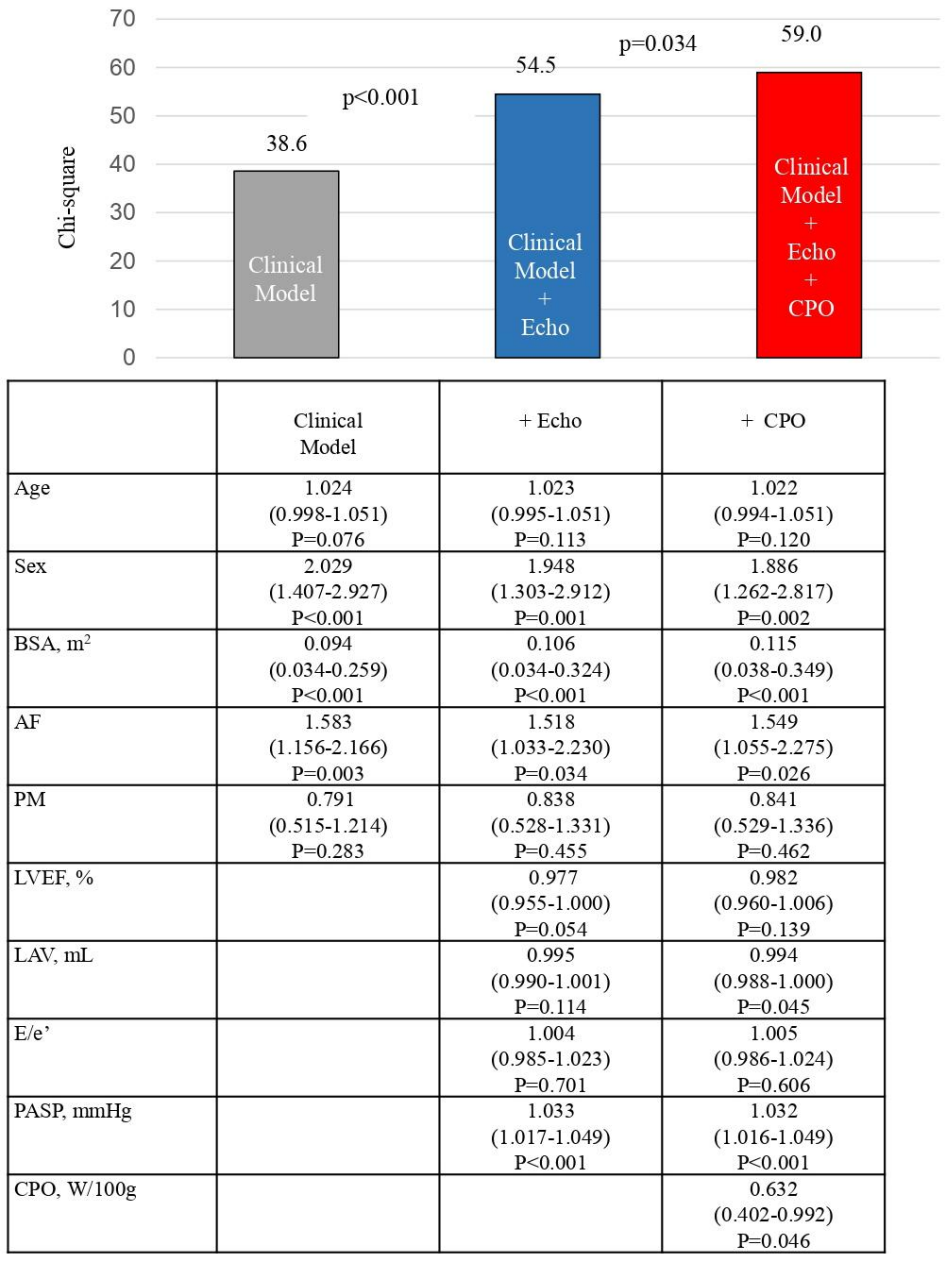
Incremental CPO values. This figure illustrates the global χ^2^ of Cox models incorporating clinical factors and echocardiographic measures.

Next, we examined the incremental prognostic value of CPO in patients with preserved LVEF after TAVI. A model based on clinical factors (age, sex, BSA, presence of AF, and post-PMI) predicted the composite endpoints (χ^2^ value 38.6, *Figure 3*). The addition of echocardiographic measures (LVEF, LAV, SPAP, and E/e′ ratio) to the model significantly improved the predictive power (χ^2^ values 54.5, 38.6, *P* < 0.001). The prognostic effect was further improved by the addition of CPO (χ^2^ value 59.0 vs. 54.3, *P* = 0.034).

### Univariable and multivariable logistic regression analyses in continuous format (lowest CPO)

Univariable logistic regression analysis showed that being male, PPM, MR (≥3°), TR (≥3°), and AF, and higher BSA, lower LVEF, larger LAV and LVEDV, lower eGFR, and higher NT-pro BNP levels were significantly associated with the lowest CPO group, using the continuous variable format (*Table 4*). Multivariable logistic regression analysis revealed that a lower LVEF, larger LAV, and PPM were independently associated with the lowest CPO group (odds ratio = 0.931, 1.020, and 1.750, *P* < 0.001, <0.001, and *P* = 0.033, respectively; *Table 4*).

**Table 4:** Uni- and multivariable logistic regression analyses for predicting the lower CPO group

	Univariable model		Multivariable model	
Variables	Odds ratio (95% CI)	*P* value	Odds ratio (95% CI)	*P* value
Sex, male	1.772 (1.373–2.288)	<0.001	1.310 (0.790–2.170)	0.296
Age, years	0.996 (0.971–1.020)	0.748	0.988 (0.955–1.020)	0.479
BSA, m^2^	2.980 (1.290–6.890)	0.011	0.372 (0.083–1.660)	0.195
LVEF, %	0.928 (0.904–0.953)	<0.001	0.931 (0.901–0.963)	<0.001
E/e′	1.010 (0.996–1.030)	0.137	0.985 (0.961–1.010)	0.230
LAV, mL	1.020 (1.020–1.030)	<0.001	1.020 (1.010–1.030)	<0.001
LVEDV, mL	1.020 (1.010–1.020)	<0.001	1.010 (0.999–1.020)	0.086
SPAP, mmHg	1.030 (1.010–1.050)	<0.001	1.010 (0.989–1.040)	0.294
PM	0.816 (0.527–1.260)	0.359	0.960 (0.562–1.640)	0.880
PPM	1.760 (1.200–2.590)	0.004	1.750 (1.050–2.910)	0.033
PVL ≥ 2°	1.760 (0.979–3.170)	0.059	1.110 (0.543–2.260)	0.777
MR ≥ 3°	2.570 (1.500–4.390)	<0.001	1.250 (0.642–2.420)	0.516
TR ≥ 3°	2.730 (1.630–4.560)	<0.001	1.450 (0.716–2.920)	0.304
Hb, g/dL	1.060 (0.949–1.190)	0.296	1.130 (0.979–1.310)	0.093
eGFR 10 mL/min/1.73 m^2^	0.895 (0.841–0.952)	<0.001	0.963 (0.871–1.060)	0.459
Log NT-pro BNP, pg/mL	1.880 (1.450–2.430)	<0.001	1.210 (0.774–1.890)	0.404
AF	1.810 (1.270–2.560)	<0.001	0.677 (0.402–1.140)	0.144

BSA, body area; LVEF, left ventricular ejection fraction; LAV, left atrial volume; LVEDV, left ventricular end-diastolic volume; SPAP, systolic pulmonary artery pressure; PM, pacemaker; PPM, prosthesis-patient mismatch; PVL, paravalvular leakage; MR, mitral regurgitation; TR, tricuspid regurgitation; Hb, haemoglobin; eGFR, estimated glomerular filtration rate; AF, atrial fibrillation.

## Discussion

The main findings of this study are as follows: (i) CPO was independently associated with an increased risk of all-cause mortality, (ii) CPO provided an independent and incremental prognostic effect for composite endpoints over clinical and established echocardiographic markers in patients with preserved LVEF after TAVI, and (iii) LV contractile function, LA size, and PPM were associated with the lowest CPO group.

### CPO and outcomes in patients with preserved LVEF after TAVI

The CPO is a comprehensive indicator of cardiac performance that incorporates both pressure and flow components. Previous studies have suggested that the prognostic effects of CPO can be studied in patients with HFpEF, heart failure with reduced EF, cardiogenic shock, and acute myocardial infarction.^9–11,14–17^ A recent study demonstrated that CPO-normalized LV mass has independent and incremental value for predicting adverse events in patients with HFpEF,^10^ however, the effectiveness of CPO in the risk stratification of patients with valvular heart disease and post-operative valvular disease has not been reported. To our knowledge, this study is the first to investigate whether CPO normalized by LV mass is associated with adverse outcomes in patients with preserved LVEF after TAVI. Previous studies have identified several prognostic indicators in patients after TAVI, including pulmonary hypertension, left atrial dilatation, AF, paravalvular leakage, and PPM.^18–24^ Our study showed that CPO was an independent prognostic factor in multivariable Cox regression analysis, in addition to the factors stated above. Gotzmann *et al*. described an increase in systolic BP immediately after TAVI that was unrelated to a change in LVEF.^25^ Increased BP is associated with increased CO and predicts better clinical outcomes.^26^ Additionally, SVi after TAVI is an independent predictor of adverse events in patients with low-gradient severe AS and is a better predictor than SVi before TAVI.^27^ These findings suggest that patients with an LV flow reserve that increases CO while resisting the increase in BP after TAVI have a better prognosis. In addition, the impact of LV mass should be considered. AS induces pressure overload of the LV and results in LV hypertrophy (LVH) due to myocyte degeneration and fibrosis. A previous study showed that echocardiographic LVH was not associated with poor prognosis.^28^ The reason for this may be the effect of the regression of LVM after TAVI.^29,30^ On the other hand, cardiac amyloidosis is a factor that causes LVH in AS. The coexistence rate of AS and cardiac amyloidosis among patients referred for TAVI ranges from 9–16%.^31,32^ These findings suggested differences in myocardial properties and function in the LVH. We consider CPO as an indicator that reflects these findings.

### Association between lower CPO and cardiac function

A previous study showed that patients with HFpEF and a lower CPO reserve had poorer biventricular systolic function and impaired chronotropic response during exercise.^11^ Our study showed that patients with preserved LVEF after TAVI in the lowest CPO group had a lower LVEF and larger LAV and PPM. AS induces LV pressure overload. This process causes LV dilatation, reduced systolic function, pulmonary hypertension, right ventricular dysfunction, MR, and TR.^33^ Despite improvements in cardiac function after TAVI, patients continue to experience cardiac damage. Additionally, PPM was associated with incomplete regression of LVH after TAVI.^34^ Our group demonstrated an association between PPM and haemodynamic changes during exercise. CO tended to be lower, and SPAP was higher in patients with PPM than those without PPM, both at rest and during exercise.^35^ We considered that patients with preserved LVEF after TAVI in the lowest CPO group had impaired LV contractility, diastolic function, and PPM.

### Limitations

This study had several limitations. First, this was a single-centre, observational, retrospective study, although long-term follow-up data were available. Therefore, inherent bias cannot be excluded in this study type. Thus, these results should be validated in multicentre prospective studies with larger populations. Secondly, patients who died within 30 days of TAVI were excluded because of possible procedure-related causes. Therefore, deaths unrelated to the procedure may occur within 30 days of TAVI. Thirdly, in some of cases, residual pressure gradients existed between the LV and aorta after TAVI. We considered that CPO may not be an accurate reflection of LV workload if residual pressure gradients exist between the LV and aorta.

## Conclusion

Our findings suggest that CPO is independently and incrementally associated with adverse outcomes, particularly all-cause mortality, and surpasses traditional clinical and echocardiographic markers in patients with preserved LVEF after TAVI. This study indicates that CPO is significantly associated with a poor prognosis in patients with preserved LVEF following TAVI.

## Data Availability

The data underlying this article were provided by St. Marianna University Hospital. Data will be shared on request to the corresponding author with permission of St. Marianna University Hospital.

## Lead author biography

Daisuke Miyahara is a cardiologist at St Marianna University School of Medicine in Kawasaki, Japan. His research interests include exercise stress echocardiography, structured heart disease, chronic heart failure, and cardiomyopathies.
